# Comparison of two dependent within subject coefficients of variation to evaluate the reproducibility of measurement devices

**DOI:** 10.1186/1471-2288-8-24

**Published:** 2008-04-22

**Authors:** Mohamed M Shoukri, Dilek Colak, Namik Kaya, Allan Donner

**Affiliations:** 1 Department of Epidemiology and Biostatistics, Schulich School of Medicine, University of Western Ontario, London, Ontario, Canada; 2 Department of Biostatistics and Epidemiology, King Faisal Specialist Hospital and Research Centre, Riyadh, Saudi Arabia; 3 Department of Genetics, King Faisal Specialist Hospital and Research Centre, Riyadh, Saudi Arabia

## Abstract

**Background:**

The within-subject coefficient of variation and intra-class correlation coefficient are commonly used to assess the reliability or reproducibility of interval-scale measurements. Comparison of reproducibility or reliability of measurement devices or methods on the same set of subjects comes down to comparison of dependent reliability or reproducibility parameters.

**Methods:**

In this paper, we develop several procedures for testing the equality of two dependent within-subject coefficients of variation computed from the same sample of subjects, which is, to the best of our knowledge, has not yet been dealt with in the statistical literature. The Wald test, the likelihood ratio, and the score tests are developed. A simple regression procedure based on results due to Pitman and Morgan is constructed. Furthermore we evaluate the statistical properties of these methods via extensive Monte Carlo simulations. The methodologies are illustrated on two data sets; the first are the microarray gene expressions measured by two plat- forms; the Affymetrix and the Amersham. Because microarray experiments produce expressions for a large number of genes, one would expect that the statistical tests to be asymptotically equivalent. To explore the behaviour of the tests in small or moderate sample sizes, we illustrated the methodologies on data from computer-aided tomographic scans of 50 patients.

**Results:**

It is shown that the relatively simple Wald's test (WT) is as powerful as the likelihood ratio test (LRT) and that both have consistently greater power than the score test. The regression test holds its empirical levels, and in some occasions is as powerful as the WT and the LRT.

**Conclusion:**

A comparison between the reproducibility of two measuring instruments using the same set of subjects leads naturally to a comparison of two correlated indices. The presented methodology overcomes the difficulty noted by data analysts that dependence between datasets would confound any inferences one could make about the differences in measures of reliability and reproducibility. The statistical tests presented in this paper have good properties in terms of statistical power.

## Background

An extensive literature has been developed on procedures for testing the equality of two or more independent coefficients of variation as measures of reproducibility [3-5]. Their work shows that likelihood-based methods such as the likelihood ratio (LR) test, score test, and tests based on the method of generalized statistics developed by Weerahandi [6], provide efficient procedures for comparing coefficient of variations (CV) in univariate normal populations or from independent samples. However, there are situations where comparing CVs from related samples should be considered. Typical situation is when two instruments are used to measure the same set of subjects, and each subject is repeatedly measured by the same instrument. We shall explain in the methods section the reason why the within-subject coefficient of variation (WSCV) is a more appropriate measure of reproducibility than the CV. Many authors use the terms reliability and reproducibility interchangeably [7-9]; however we believe that they are conceptually different. The reliability is the degree of closeness of the repeated observation on the same subject under the same experimental conditions, so the instrument is always the same. The Intra-class correlation coefficient (ICC) is commonly used as a measure of reliability. It is calculated as the ratio between subjects variance to the total variance. Therefore, the larger the heterogeneity among the subjects, with lower or equal random error the easier it is to differentiate among subjects. In other words, the ICC measures how distinguishable the subjects are. On the other hand, reproducibility determines the degree of closeness of the repeated observations made on the same subject either by the same instrument or different instruments. There is a wide debate among statisticians and psychometricians related to the choice of appropriate measures of reliability and reproducibility. We refer the interested reader to [10,11]. The main focus of our paper is on the reproducibility parameter.

An important application from molecular biology research in which correlated/dependent reproducibility coefficients are compared is when microarray technologies are compared in terms of reproducibility of gene expression measurements. DNA Microarrays are powerful technologies which make it possible to study genome-wide gene expressions and are extensively used in biological research. As the technology evolves rapidly a number of different platforms became available, which introduces some challenges for researchers to know which technology is best suited for their needs. There have been various studies that directly compared the performance of one platform with another in terms of cross-platform comparability and agreement of gene expression results. However the results of these studies are conflicting: some demonstrate concordance, others discordance between technologies [12-17]. Thus one needs to take into consideration the accuracy and reproducibility of different types of microarrays when allocating the laboratory resources for future experiments. The key factors for selecting an appropriate platform are (1) Intra-assay reproducibility, and (2) the degree of cross-platform agreement [18]. The concordance among microarray platforms would allow researchers to directly compare their measurements and perform meta-analyses.

Most of the microarray reliability or reproducibility and cross-platform studies use Pearson's correlation, as an index of reproducibility or agreement. However, it has long been recognized that application of procedures such as the paired t-test and Pearson's correlation are not appropriate tools for measuring agreement between measuring devices [19,20]. Rather, indices such as the intra-class correlation coefficient [21] and the within- subject coefficient of variation should be used as measures of reproducibility. It has also been demonstrated that the within-subject coefficient of variation is very useful in assessing instrument reproducibility [8,22].

The main focus of this paper is to develop several procedures for testing the equality of two dependent within-subject coefficients of variation computed from the same sample of subjects, which is, to the best of our knowledge, has not been dealt with in the statistical literature, and to evaluate the statistical properties of these methods via extensive Monte Carlo simulation. We propose two approaches; one is likelihood based (LRT, Wald, and Score test), and the other is a regression based approach coined as PM test. After evaluating the statistical properties (power and empirical level of significance) of these tests using Monte Carlo simulation, the methodology is illustrated on data from two biomedical studies.

## Methods

### Likelihood based methodology

Suppose that we are interested in comparing the reproducibility of two instruments. Let *x*_*ijl *_be the *jth *measurement of the *ith *subject by the *lth *instrument, *j *= 1,2,... *m*_*l*_, *i *= 1,2,... *n*, and *l *= 1, 2. To evaluate the WSCV we consider the one-way random effects model

(1) *x*_*ijl *_= *μ*_*l *_+ *b*_*i *_+ *e*_*ijl *_

where *μ*_*l *_is the mean value of measurements made by the *lth *instrument, *b*_*i *_are independent random subject effects with *b*_*i *_~ *N*(0, $\sigma_{b}^{2}$), and *e*_*ijl *_are independent *N*(0, $\sigma_{l}^{2}$). Many authors have used the intra-class correlation coefficient (ICC), *ρ*_*l *_defined by the ratio $\rho_{l}=\sigma_{b}^{2}/\left(\sigma_{b}^{2}+\sigma_{l}^{2}\right)$ as measure of reproducibility/reliability [18,23]. Quan and Shih [8] argued that *ρ*_*l *_is study-population based since it involves between-subject variation. Meaning that the more heterogeneity in the population, the larger the *ρ*_*l*_. Alternatively, they proposed the within-subject coefficient of variation (WSCV) *θ*_*l *_= *σ*_*l*_/*μ*_*l *_as a measure of reproducibility. It determines the degree of closeness of repeated measurements taken on the same subject either by the same instruments or on different occasions under the same conditions. It is clear that, the smaller the WSCV, the better the reproducibility. We distinguish the WSCV from the coefficient of variation CVl=(σb2+σl2)12/μl since *CV*_*l *_involves $\sigma_{b}^{2}$ in the numerator and similar to *ρ*_*l *_is population based. Therefore, more heterogeneity in the population would result in a large value of *CV*_*l*_. For that reason we shall focus our work on the WSCV rather than the CV. We also note that there is an inverse relationship between the ICC (*ρ*_*l*_) and the corresponding within subject variance $\sigma_{l}^{2}$. Clearly, larger values of ICC (higher reliability) would be associated with smaller WSCV (better reproducibility). The focus of this paper is on aspects of statistical inference on the difference between two correlated WSCV. The inferential procedure depends on the multivariate normality of the measurements and is mainly likelihood based. The following set-up is to facilitate the construction of the likelihood function.

Let

$$X_{i}={\left(X_{i1},X_{i2},.......X_{im_{1}},X_{i,m_{1}+1},X_{i,m_{1}+2},....,X_{i,m_{1}+m_{2}}\right)}^{'}$$

denote the measurements on the *i*^*th *^subject, *i *= 1,2,....,*n *where $X_{i1},X_{i2},....,X_{im_{1}}$ are the *m*_1 _measurements obtained by the first method (platform), $X_{im_{1}+1},X_{im_{1}+2},....,X_{im_{1}+m_{2}}$ are the *m*_2 _measurements obtained the second method (platform). We assume that *X*_*i *_~ *N*(*μ*, Σ), where $\mu^{T}=(\mu_{1}1_{m_{1}}^{T},\mu_{2}1_{m_{2}}^{T})$ and,

(2) $$\Sigma =\left[\begin{array}{cc} \sigma_{1}^{2}I_{m_{1}}+\frac{\rho_{1}}{1-\rho_{1}}\sigma_{1}^{2}J_{m_{1}} & \rho_{12}\sigma_{1}\sigma_{2}J_{m_{1}xm_{2}}\\ \rho_{12}\sigma_{1}\sigma_{2}J_{m_{1}xm_{2}} & \sigma_{2}^{2}I_{m_{2}}+\frac{\rho_{2}}{1-\rho_{2}}\sigma_{2}^{2}J_{m_{2}} \end{array}\right]$$

In these expressions 1_*k *_is a column vector with all *k *elements equal to 1, *I*_*k *_is a *k *× *k *identity matrix and *J*_*k *_and *J*_*kxt *_are *k *× *k *and *k *× *t *matrices with all the elements equal to 1. Thus the model assumes that the *m*_1 _observations taken by the first platform have common mean *μ*_1_, common variance $\sigma_{1}^{2}$, and common intra-class correlation *ρ*_1_, whereas the *m*_2 _measurements taken by the second platform have common mean *μ*_2_, common variance $\sigma_{2}^{2}$, and common intra-class correlation *ρ*_2_. Moreover, *ρ*_12 _denotes the interclass correlation between any pair of measurements *x*_*ij *_(*j *= 1,2,... *m*_1_) and $x_{im1+t}\left(t=1,2,\ldots m_{2}\right)$, and also assumed constant across all subjects in the population.

For the *l*^*th *^method, the WSCV, which will be denoted as *θ*_*l *_in the remainder of the paper is defined as

*θ*_*l *_= *σ*_*l*_/*μ*_*l*_,   *l *= 1, 2.

Our primary aim is to develop and evaluate methods of testing *H*_0_:*θ*_1 _= *θ*_2 _taking into account dependencies induced by a positive value of *ρ*_12_. We restrict our evaluation to reproducibility studies having *m*_1 _= *m*_2 _= *m*.

### Methods for testing the null hypothesis

#### Wald test (WT)

If *X*_1_, *X*_2_,.... *X*_*n *_is a sample from the above multivariate normal distribution, then the *log*-likelihood function *l*, as a function of *ψ *= (*μ*_1_, *μ*_2_, $\sigma_{1}^{2}$, $\sigma_{2}^{2}$, *ρ*_1_, *ρ*_2_, *ρ*_12_) is given by:

(3) $$-2L=Q+nm\log\left(\sigma_{1}^{2}\sigma_{2}^{2}\right)-n\log\left(\left(1-\rho_{1}\right)\left(1-\rho_{2}\right)\right)+n\log w$$

where,

*w *= *u*_1_*u*_2 _- *m*^2 ^$\rho_{12}^{2}$,

*u*_*l *_= 1 + (*m *- 1)*ρ*_*l*_, *l *= 1, 2 and,

$$\begin{array}{c} Q=\frac{S_{1}^{2}}{\sigma_{1}^{2}}+\frac{m\left(1-\rho_{1}\right)u_{2}}{w\sigma_{1}^{2}}\sum_{i=1}^{n}{\left({\bar{x}}_{i1}-\mu_{1}\right)}^{2}+\frac{S_{2}^{2}}{\sigma_{2}^{2}}+\frac{m\left(1-\rho_{2}\right)u_{1}}{w\sigma_{2}^{2}}\sum_{i=1}^{n}{\left({\bar{x}}_{i2}-\mu_{2}\right)}^{2}\\ -\frac{2m^{2}\rho_{12}}{w\sigma_{1}\sigma_{2}}{\left(\left(1-\rho_{1}\right)\left(1-\rho_{2}\right)\right)}^{1/2}\sum_{i=1}^{n}\left({\bar{x}}_{i1}-\mu_{1}\right)\left({\bar{x}}_{i2}-\mu_{2}\right) \end{array}$$

From [24] the conditions {1 + (*m *- 1)*ρ*_1_}{1 + (*m *- 1)*ρ*_2_} > *m*^2 ^$\rho_{12}^{2}$ and -1/(*m *- 1) <*ρ*_*l *_< 1 must be satisfied for the likelihood function to be a sample from a non-singular multivariate normal distribution.

The summary statistics given in (3) are defined as:

$$\begin{array}{ll} {\bar{x}}_{ij}=\sum_{k=1}^{m}x_{ijk}/m & i=1,2,\ldots n;j=1,2\\ S_{j}^{2}=\sum_{i=1}^{n}\sum_{k=1}^{m}{\left(x_{ijk}-{\bar{x}}_{ij}\right)}^{2} & \end{array}$$

The maximum likelihood estimates (MLE) for *μ*_*l *_and $\sigma_{l}^{2}$ are given respectively by ${\overset{⌢}{\mu }}_{l}={\bar{x}}_{l},{\overset{⌢}{\sigma }}_{l}^{2}=S_{l}^{2}/n\left(m-1\right)$, where ${\bar{x}}_{l}=\frac{1}{n}\sum_{i=1}^{n}{\bar{x}}_{ij}$ and *l *= 1, 2. Clearly, ${\hat{\sigma }}_{l}^{2}$ exists for values of *m *> 1. Therefore we shall assume that *m *> 1 throughout this paper. From [24], we obtain ${\hat{\rho }}_{1}$ and ${\hat{\rho }}_{2}$ by computing Pearson's product-moment correlation over all possible pairs of measurements that can be constructed within platforms 1 and 2 respectively, with ${\hat{\rho }}_{12}$ similarly obtained by computing this correlation over the *nm*^2 ^pairs (*x*_*ij*_, *x*_*i*_,_*m*+*l*_).

The WT of *H*_0_:*θ*_1 _= *θ*_2 _requires the evaluation of variance of ${\overset{⌢}{\theta }}_{l}$, *l *= 1, 2, and $\mathrm{cov}\left({\overset{⌢}{\theta }}_{1},{\overset{⌢}{\theta }}_{2}\right)$. To obtain these values we use elements of Fisher's information matrix, along with the delta method [26,27]. On writing:

*ψ *= (*ψ*_1_, *ψ*_2_)',*ψ*_1 _= (*μ*_1_, *μ*_2_)', *and *$\psi_{2}={\left(\sigma_{1}^{2},\sigma_{2}^{2},\rho_{1},\rho_{2},\rho_{12}\right)}^{\prime }$, the Fisher's information matrix *I *= -*E*⌊∂^2^*l*/∂*ψ*∂*ψ*'⌋ has the following structure:

(4) $$I=\left[\begin{array}{cc} I_{11} & O\\ O & I_{22} \end{array}\right].$$

This is based on a result from [26] (page 239) indicating that, *I*_12 _= $I_{21}^{'}$ = -*E*(∂^2^*l*/∂*ψ*_1_∂*ψ*'_2_) = 0. Therefore, from the asymptotic theory of maximum likelihood estimation we have:

$$I_{11}^{-1}=\left[\begin{array}{cc} \mathrm{var}\left({\overset{⌢}{\mu }}_{1}\right) & \mathrm{cov}\left({\overset{⌢}{\mu }}_{1},{\overset{⌢}{\mu }}_{2}\right)\\ \mathrm{cov}\left({\overset{⌢}{\mu }}_{1},{\overset{⌢}{\mu }}_{2}\right) & \mathrm{var}\left({\overset{⌢}{\mu }}_{2}\right) \end{array}\right]$$

And the elements of *I*_22 _are given in the Appendix.

The elements of $I_{22}^{-1}$ are the asymptotic variance- covariance matrix of the maximum likelihood estimators of the covariance parameters. Inverting Fisher's information matrices we get:

(5) $$\mathrm{var}\left({\overset{⌢}{\mu }}_{l}\right)=\frac{\sigma_{l}^{2}}{nm\left(1-\rho_{l}\right)}\left[1+\left(m-1\right)\rho_{l}\right].$$

Applying the delta method [27], we can show, to the first order of approximation that:

(6) $$\begin{array}{cc} \mathrm{var}\left({\overset{⌢}{\sigma }}_{l}\right)\approx \sigma_{l}^{2}/2n\left(m-1\right). & l=1,2 \end{array}$$

The maximum likelihood estimator of *θ*_*l *_is ${\hat{\theta }}_{l}=\frac{{\hat{\mu }}_{l}}{{\hat{\sigma }}_{l}}$. Again, by application of the delta method, we can show to the first order of approximation that:

(7) $$\mathrm{var}\left({\overset{⌢}{\theta }}_{l}\right)\approx \frac{\theta_{l}^{4}\left[1+\left(m-1\right)\rho_{l}\right]}{nm\left(1-\rho_{l}\right)}+\frac{\theta_{l}^{2}}{2n\left(m-1\right)},$$

as was shown by Quan and Shih [8].

Again using the delta method we show approximately that:

(8) $$\mathrm{cov}\left({\overset{⌢}{\theta }}_{1},{\overset{⌢}{\theta }}_{2}\right)\approx \frac{2\theta_{1}^{2}\theta_{2}^{2}\rho_{12}}{n\sqrt{\left(1-\rho_{1}\right)\left(1-\rho_{2}\right)}}.$$

From [28] we apply the large sample theory of maximum likelihood to establish that:

(9) $$Z=\frac{{\hat{\theta }}_{1}-{\hat{\theta }}_{2}}{\sqrt{\mathrm{var}({\hat{\theta }}_{1})+\mathrm{var}({\hat{\theta }}_{2})-2\mathrm{cov}({\hat{\theta }}_{1},{\hat{\theta }}_{2})}}$$

is approximately distributed under *H*_0 _as a standard normal deviate. The denominator of Z is the standard error of ${\hat{\theta }}_{1}-{\hat{\theta }}_{2}$ and is denoted by *SE *${\hat{\theta }}_{1}-{\hat{\theta }}_{2}$. Since the standard error of ${\hat{\theta }}_{1}-{\hat{\theta }}_{2}$ contains unknown parameters, its maximum likelihood estimate $\hat{S}E({\hat{\theta }}_{1}-{\hat{\theta }}_{2})$ is obtained by substituting ${\hat{\theta }}_{l}$ for *θ*_*l*_, ${\tilde{\rho }}_{l}$ for *ρ*_*l *_and ${\tilde{\rho }}_{12}$ for *ρ*_12_. Moreover, we may construct an approximate (1-*α*)100% confidence interval on (*θ*_1 _- *θ*_2_) given as:

${\hat{\theta }}_{1}-{\hat{\theta }}_{2}\pm z_{\alpha /2}\hat{S}E({\hat{\theta }}_{1}-{\hat{\theta }}_{2})$, where *z*_*α*/2 _is the (1-*α*/2)100% cut-off point of the standard normal distribution.

#### Likelihood ratio test (LRT)

An LRT of *H*_0 _: *θ*_1 _= *θ*_2 _was developed numerically, and computed by first setting *μ*_*l *_= *σ*_*l*_/*θ*_*l*_, *l *= 1,2 in Equation (3), and then adopting the following algorithm:

1- Set *μ*_*l *_= *σ*_*l*_/*θ*_*l*_, *l *= 1,2 in Equation (3), thereafter;

2- Set *θ*_1 _= *θ*_2 _= *θ *in (3)

3- Minimize the resulting expression with respect to all six parameters (*σ*_1_, *σ*_2_, *ρ*_1_, *ρ*_2_, *ρ*_12_, *θ*) and;

4- Subtract the minimum from the minimum of -2*L *as computed over all seven parameters (*σ*_1_, *σ*_2_, *ρ*_1_, *ρ*_2_, *ρ*_12_, *θ*_1_, *θ*_2_) in the model.

It then follows from standard likelihood theory that the resulting test statistic is approximately chi-square distributed with 1 degree of freedom under H_0_.

#### Score test

One of the advantages of likelihood based inference procedure is that in addition to the WT and the LRT "Rao's score test" can also be readily developed. The motivation for it is that it can sometimes be easier to maximize the likelihood function under the null hypothesis than under the alternative hypothesis. A standard procedure for performing the score test of *H*_0 _: *θ*_1 _= *θ*_2 _is to set *θ*_2 _= *θ*_1 _+ Δ, so that the null hypothesis is equivalent to *H*_0 _: Δ = 0, where Δ is unrestricted. Replacing *μ*_*l *_by *σ*_*l*_/*θ*_*l*_, the log-likelihood function *L *is then independent of *μ*_*l*_.

Let *L *= *L*(Δ; *ψ*^•^) = *L*(Δ; *θ*_1_, *σ*_1_, *σ*_2_, *ρ*_1_, *ρ*_2_, *ρ*_12_) and $l_{1}=\frac{\partial L}{\partial \Delta },l_{2}=\frac{\partial L}{\partial \psi^{*}}$.

From [28] the score statistic is given by:

$$S={\dot{l}}_{1}^{T}A_{1•2}^{-1}{\dot{l}}_{1},$$

where

(10) $$l_{1}^{•}=\frac{\partial L}{\partial \Delta }\underset{\Delta =0}{|}=\frac{nm}{\hat{w}{\hat{\theta }}_{1}^{2}}\left[{\hat{\mu }}_{1}\left(1-{\hat{\rho }}_{2}\right)\left(\frac{{\hat{\theta }}_{1}-{\hat{\theta }}_{2}}{{\hat{\theta }}_{1}{\hat{\theta }}_{2}}\right)\right]$$

and $A_{1•2}=A_{11}-A_{12}A_{22}^{-1}A_{21}$. The matrices on the right hand side of *A*_1•2 _are obtained from partitioning the Fisher's information matrix A so that $A=\left(\begin{array}{cc} A_{11} & A_{12}\\ A_{21} & A_{22} \end{array}\right)$ where $A_{11}=E\left(-\frac{\partial^{2}l}{\partial \Delta^{2}}\right),A_{12}=A_{21}^{T}=E\left(-\frac{\partial^{2}l}{\partial \Delta \partial \psi^{*}}\right)$, and $A_{22}=E\left(-\frac{\partial^{2}l}{\partial \psi^{*}\partial \psi^{*T}}\right)$ with all the matrices on the right hand side of *A*_1•2 _evaluated at Δ = 0. When an estimator other than the MLE is used for the nuisance parameters ψ*, provided that the estimator ${\hat{\psi }}^{*}$ is $\sqrt{n}$ consistent, it was shown that the asymptotic distribution of *S *is that of a chi-square with 1 degree of freedom [29,30].

The score test has been applied in many situations and has been proven to be locally powerful. Unfortunately, the inversion of *A*_1•2 _is quite complicated and we cannot obtain a simple expression for *S *that can be easily used. Moreover, we have also found through extensive simulations that while the score test holds its levels of significance, it is less powerful than LRT and WT across all parameter configurations. We therefore focus our subsequent discussion of power to LRT and WT.

#### Regression test

Pitman [1] and Morgan [2] introduced a technique to test the equality of variances of two correlated normally distributed random variables. It is constructed to simply test for zero correlation between the sums and differences of the paired data. Bradley and Blackwood [31] extended Pitman and Morgan's idea to a regression context that affords a simultaneous test for both the means and the variances. The test is applicable to many paired data settings, for example, in evaluating the reproducibility of lab test results obtained from two different sources. The test could also be used in repeated measures experiments, such as in comparing the structural effects of two drugs applied to the same set of subjects. Here we generalize the results of Bradley and Blackwood to establish the simultaneous equality of means and variances of two correlated variables, implying the equality of their coefficients of variations.

Let ${\bar{X}}_{ij}=\sum_{k=1}^{m}X_{ijk}/m$, and define $d_{i}={\bar{X}}_{i1}-{\bar{X}}_{i2}$, and $s_{i}={\bar{X}}_{i1}+{\bar{X}}_{i2}$.

Direct application of the multivariate normal theory shows that the conditional expectation of *d*_*i *_on *s*_*i *_is linear [32]. That is

(11) *E*(*d*_*i *_| *s*_*i*_) = *α *+ *βs*_*i*_,

where

(11.a) $$\alpha =\left(\mu_{1}-\mu_{2}\right)-\left(\mu_{1}+\mu_{2}\right)\left(\sigma_{1}^{2}-\sigma_{2}^{2}\right)k$$

(11.b) $$\beta =\left(\sigma_{1}^{2}-\sigma_{2}^{2}\right)k$$

where

$k^{-1}=\sigma_{1}^{2}{\left(1-\rho_{1}\right)}^{-1}\left(1+\left(2m-1\right)\rho_{1}\right)+\sigma_{2}^{2}{\left(1-\rho_{2}\right)}^{-1}\left(1+\left(2m-1\right)\rho_{2}\right)$ is strictly positive.

The proof is straightforward and is therefore omitted.

It can be shown then from direct application of the multivariate normal theory that the conditional expectation (11) is linear, and does not depend on the parameter *ρ*_12_.

From (11.a) and (11.b), it is clear that *α *= *β *= 0 if and only if *μ*_1 _= *μ*_2 _and *σ*_1 _= *σ*_2 _simultaneously. Therefore, testing the equality of two correlated coefficients of variations is equivalent to testing the significance of the regression equation (11). From the theory of least squares, if we define:

$\text{TSS}=\sum_{i=1}^{n}{\left(d_{i}-\bar{d}\right)}^{2},RSS={\hat{\beta }}_{1}^{2}\sum_{i=1}^{n}{\left(s_{i}-\bar{s}\right)}^{2}$ and EMS = (*TSS *- *RSS*)/(*n *- 2),

the hypothesis *H*_0 _: *α *= *β *= 0 is rejected when *RSS/EMS *exceeds *F*_*v*,1,(*n*-2)_, the (1 - *v*) 100% percentile value of the *F*-distribution with 1 and *(n-2) *degrees of freedom [32].

## Results

### Simulation

The theoretical properties of the test procedures discussed thus far are largely intractable in finite samples. We therefore took a Monte Carlo study to determine the levels of significance and powers of these tests over a wide range of parameter values. For this study we generated observations from a multivariate normal distribution with covariance structure defined as in (2). Simulations were performed using programs written in MATLAB (The Math. Works, Inc., Natic, MA).

The parameters of the simulation included the total number of subjects (*n*), the number of replications (*m*_1 _= *m*_2 _= *m*), and various values of (*θ*_1_, *θ*_2_, *ρ*_1_, *ρ*_2_, *ρ*_12_). For each of 2000 independent runs of an algorithm constructed to generate observations from multivariate normal distribution, we estimated the true level of significance and power of the LRT, Wald, Score and PM tests using a nominal level of significance 5% (two sided) for various combinations of parameters.

Tables 1 and 2 report the empirical significance levels based on *2000 *simulated datasets for four (WT, Score, LRT and PM) procedures for sample size of *n = 50 *and *n = 100*, respectively. It is seen that all procedures provide satisfactory significance levels at all parameter values examined. The empirical significance levels for smaller sample sizes (*n *= 10, 20, and 30) were also estimated. All test procedures provided empirical levels that are very close to the 5% nominal level (data not shown).

**Table 1:** Empirical significance levels based on 2000 runs at nominal level 5% (two sided) for testing *θ*_1 _= *θ*_2 _= 0.15 using the LRT, Wald, Score and PM for *n *= 50 subjects and m replicates, ρ_1 _= ρ_2 _= ρ.

n = 50	*ρ *= 0.4			ρ = 0.6			ρ = 0.7		
ρ_12_	0.1	0.2	0.3	0.1	0.3	0.5	0.1	0.4	0.6
m = 2									
Wald	0.049	0.048	0.050	0.051	0.050	0.049	0.046	0.052	0.048
Score	0.057	0.051	0.053	0.055	0.055	0.058	0.051	0.058	0.054
PM	0.052	0.050	0.047	0.050	0.049	0.048	0.053	0.052	0.051
LRT	0.051	0.051	0.052	0.052	0.051	0.049	0.050	0.048	0.050
m = 3									
Wald	0.048	0.046	0.049	0.052	0.049	0.050	0.048	0.047	0.049
Score	0.056	0.053	0.055	0.058	0.054	0.051	0.054	0.047	0.051
PM	0.053	0.052	0.050	0.049	0.049	0.052	0.052	0.050	0.052
LRT	0.050	0.047	0.051	0.053	0.047	0.051	0.049	0.045	0.048
m = 5									
Wald	0.048	0.049	0.052	0.045	0.049	0.050	0.050	0.049	0.046
Score	0.054	0.050	0.051	0.051	0.053	0.054	0.049	0.048	0.056
PM	0.050	0.052	0.050	0.048	0.051	0.049	0.053	0.052	0.047
LRT	0.051	0.051	0.050	0.048	0.050	0.049	0.049	0.050	0.044

**Table 2:** Empirical significance levels based on 2000 runs at nominal level 5% (two sided) for testing *θ*_1 _= *θ*_2 _= 0.15 using the LRT, Wald, Score and PM for *n *= 100 subjects and m replicates, ρ_1 _= ρ_2 _= ρ.

n = 100	ρ = 0.4			ρ = 0.6			ρ = 0.7		
ρ_12_	0.1	0.2	0.3	0.1	0.3	0.5	0.1	0.4	0.6
m = 2									
Wald	0.049	0.048	0.051	0.049	0.050	0.045	0.046	0.050	0.051
Score	0.050	0.056	0.056	0.053	0.055	0.049	0.051	0.057	0.056
PM	0.049	0.048	0.052	0.049	0.049	0.050	0.050	0.051	0.051
LRT	0.048	0.044	0.051	0.044	0.050	0.042	0.042	0.048	0.050
m = 3									
Wald	0.051	0.050	0.048	0.048	0.049	0.049	0.051	0.047	0.044
Score	0.051	0.049	0.048	0.050	0.054	0.053	0.057	0.052	0.056
PM	0.052	0.050	0.052	0.048	0.049	0.049	0.049	0.050	0.048
LRT	0.050	0.051	0.050	0.047	0.046	0.048	0.048	0.050	0.043
m = 5									
Wald	0.050	0.049	0.052	0.049	0.048	0.050	0.051	0.050	0.046
Score	0.053	0.052	0.054	0.054	0.053	0.051	0.052	0.053	0.052
PM	0.050	0.049	0.053	0.049	0.051	0.050	0.049	0.050	0.047
LRT	0.049	0.050	0.052	0.048	0.050	0.049	0.047	0.051	0.045

Tables 3 and 4 display empirical powers based on *2000 *simulated datasets for WT and LRT in sample sizes *n *= 30 and 50, respectively. As alluded to earlier, the score test is excluded from the power Tables 3 and 4 because its simulated empirical power values were unacceptably low (as we show in Table 5). We observe that for all parameter values that WT and LRT provide almost identical values of power (Tables 3 and 4). Thus, although the LRT shows greater power at some parameter combinations than the WT, the difference is usually less than three percentage points. We also conducted simulations to estimate the powers of the test statistics for smaller sample sizes (n = 10, and 20) (data not shown). We found that for some parameter combinations Wald and LRT provided acceptable power especially if the distance between *θ*_1 _and *θ*_2 _is large, and showed greater power than both the Score and PM tests. The power of Score test was generally very low.

**Table 3:** Empirical power based on 2000 runs for testing *θ*_1 _= *θ*_2 _using the LRT and Wald test for *n *= 30 subjects.

n = 30	(ρ_1_, ρ_2_) = (0.7,0.5) (θ_1_, θ_2_) =(0.1,0.2)			(ρ_1_, ρ_2_) = (0.6, 0.5) (θ_1_, θ_2_) = (0.15,0.2)			(ρ_1_, ρ_2_) = (0.5, 0.4) (θ_1_, θ_2_) = (0.2, 0.3)		
ρ_12_	0.2	0.3	0.4	0.2	0.3	0.4	0.1	0.2	0.3
m = 2									
Wald	0.92	0.93	0.94	0.30	0.33	0.32	0.55	0.50	0.51
LRT	0.94	0.95	0.96	0.35	0.33	0.34	0.60	0.56	0.54
m = 3									
Wald	0.99	1.00	1.00	0.55	0.56	0.54	0.79	0.80	0.79
LRT	1.00	1.00	1.00	0.57	0.56	0.55	0.82	0.83	0.83
m = 5									
Wald	1.00	1.00	1.00	0.80	0.82	0.84	0.96	0.95	0.97
LRT	1.00	1.00	1.00	0.81	0.82	0.85	0.97	0.97	0.98

**Table 4:** Empirical power based on 2000 runs for testing θ_1 _= θ_2 _using the LRT and Wald test for *n *= 50 subjects.

n = 50	(ρ_1_, ρ_2_) = (0.7,0.5) (θ_1_, θ_2_) = (0.1,0.2)			(ρ_1_, ρ_2_) = (0.6, 0.5) (θ_1_, θ_2_) = (0.15,0.2)			(ρ_1_, ρ_2_) = (0.5, 0.4) (θ_1_, θ_2_) = (0.2, 0.3)		
ρ_12_	0.2	0.3	0.4	0.2	0.3	0.4	0.1	0.2	0.3
m = 2									
Wald	0.99	0.98	0.99	0.47	0.50	0.49	0.75	0.72	0.74
LRT	0.99	0.99	0.99	0.49	0.52	0.51	0.77	0.77	0.78
m = 3									
Wald	1.00	1.00	1.00	0.76	0.77	0.78	0.94	0.95	0.95
LRT	1.00	1.00	1.00	0.79	0.78	0.79	0.95	0.95	0.96
m = 5									
Wald	1.00	1.00	1.00	0.94	0.93	0.95	1.00	0.99	1.00
LRT	1.00	1.00	1.00	0.95	0.95	0.96	1.00	0.99	1.00

**Table 5:** Empirical Power of PM, Score and Wald tests based on 2000 data sets, *n *= 50 subjects, *m *= 3 replicates.

(μ_1_, μ_2_)	(θ_1_, θ_2_)	ρ_1_	ρ_2_	ρ_12_	PM	Score	Wald
(10,10)	(0.2,0.3)	0.5	0.4	0.3	0.53	0.37	0.94
	(0.2,0.4)	0.5	0.3	0.2	0.84	0.51	0.99
(8,10)	(0.2,0.3)	0.5	0.4	0.3	0.71	0.40	0.95
	(0.2,0.4)	0.5	0.3	0.2	0.69	0.51	1.00
(6,10)	(0.2,0.3)	0.5	0.4	0.3	0.84	0.35	0.94
	(0.2,0.4)	0.5	0.3	0.2	0.99	0.54	1.0
(5,10)	(0.2,0.3)	0.5	0.4	0.3	0.91	0.40	0.95
	(0.2,0.4)	0.5	0.3	0.2	0.997	0.54	1.00

For selected parameter values, power levels of PM, Wald, and the score tests for n = 50 subjects are given in Table 5. As already mentioned, the power of the score test is generally low. We note that the power of the Wald test is quite sensitive to the distance between *θ*_1 _and *θ*_2_. We note that the equality of the means and variances implies the equality of the WSCV, but the reverse is not true. This strong assumption might explain the relatively poor performance of the PM test, particularly when the means are not well separated.

To assess the effect of non-normality on the properties of the proposed test statistics we generated data from a log-normal distribution, and evaluated the performance of the four procedures for 2000 simulated datasets. The empirical levels of the regression based PM test were quite close to the 5% nominal level, but the power was poor. However, the likelihood based procedures (Wald, LRT and Score) did not preserve their nominal levels for the majority of the parameters combinations (data not shown).

### Applications

#### Gene expression data

We illustrate the proposed methodologies by analyzing data from two biomedical studies. In the first data sets we illustrate the methodology on the gene expression measurement results of identical RNA preparations for two commercially available microarray platforms, namely, Affymerix (25-mer), and Amersham (30-mer) [14]. The RNA was collected from pancreatic PANC-1 cells grown in a serum-rich medium ("control") and 24 h following the removal of the serum ("treatment"). Three biological replicates (B1, B2, and B3) and three technical replicates (T1, T2, and T3) for the first biological replicate (B1) were produced by each platform. Therefore, for each condition (control and treatment) five hybridizations are conducted. The dataset consists of 2009 genes that are identified as common across the platforms after comparing their Gene Bank IDs, and is normalized according to the manufacturer's standard software and normalization procedures. More details concerning this dataset can be found in the original article [14].

The results presented in this section were not restricted to the group of differentially expressed genes, and we used the "control" part of the data for both technical and biological replicates. The normalized intensity values are averaged for genes with multiple probes for a given Gene ID. Hence, we have a sample size of n = 2009 genes measured three times (m = 3) by each of the two platforms (or instruments). We have used the within- gene coefficient of variation as a measure of reproducibility of a specific platform.

The results of the data analyses are summarized in Table 6. Parameter estimates for both platforms, the estimated WSCV under the null hypotheses, as well as confidence interval of the difference of the two WSCVs are given in the Table. We note that the correlation estimates remain the same under both hypotheses. Moreover, we note that the intraclass correlations (*ρ*) are quite high. Using benchmarks provided in [33], both platforms produce substantially reproducible gene expression levels. Clearly, this is due to the large heterogeneity among the genes in the data set. Application of the LRT, Wald, and the PM tests for testing the equality of two dependent WSCV show that the Amersham has significantly lower WSCV (P < 0.001) i.e. better reproducibility for both the technical and biological replicates.

**Table 6:** Microarray Gene Expression data results (n = 2009 genes, m = 3 replicates)

(a) Technical replicate				
	Affymetrix (*l *= 1)		Amersham (*l *= 2)	
	Estimate	SE	Estimate	SE

*μ*_*l*_	2759	150.5	3.74	0.22
*ρ *_*l*_	0.94	0.002	0.99	0.0003
*θ*_*l*_	0.58	0.03	0.25	0.015
*σ*_*l*_	1603	17.88	0.93	0.01
*ρ*_12 _= 0.51, T_wald _= 11.85, LRT = 122, PM = -7.89 (p < 0.001 for all tests) The estimate of the common WSCV under the null is 0.31 (SE = 0.014) 95% CI for (θ_1_-θ_2_): (0.28, 0.39)				

(b) Biological replicate				

	Affymetrix (*l *= 1)		Amersham (*l *= 2)	
	Estimate	SE	Estimate	SE

*μ*_*l*_	2819	142.6	3.43	0.18
*ρ *_*l*_	0.91	0.003	0.93	0.0025
*θ*_*l*_	0.71	0.037	0.63	0.034
*σ*_*l*_	2003.7	22.35	2.16	0.02
*ρ*_12 _= 0.50, T_wald _= 2.35, LRT= 8.56, PM = -9.04 (p < 0.02 for all tests) The estimate of the common WSCV under the null is 0.67 (SE = 0.025) 95% CI for (θ_1_-θ_2_): (0.014, 0.15)				

#### Analysis of computer aided tomographic scan measurements

Here we demonstrate the statistical methodologies of this paper on a much smaller data set than the microarray gene expression example. The data are from a study using the Computer-Aided Tomographic Scans (CAT-SCAN) of the heads of 50 psychiatric patients [20,34]. The measurements are the size of the brain ventricle relative to that of the patient's skull, and given by the ventricle-brain ratio VBR = (ventricle size/brain size) × 100. For a given scan, VBR was determined from measurements of the perimeter of the patient's ventricle together with the perimeter of the inner surface of the skull. These measurements were taken either: (i) from an automated pixel count (PIX) based on the images displayed on a television screen, or (ii) a hand-held planimeter (PLAN) on a projection of the X-ray image. Table 7 summarizes the results. Clearly all tests show that PIX has significantly lower WSCV that the PLAN (p < 0.001); that is better reproducibility.

**Table 7:** Analysis of computer-aided tomographic scan data on 50 patients via PIX or PLAN with two replicates

	PIX (*l *= 1)		PLAN (*l *= 2)	
	Estimate	SE	Estimate	SE
*μ*_*l*_	1.41	0.074	1.79	0.056
*ρ *_*l*_	0.99	0.002	0.73	0.066
*θ*_*l*_	0.028	0.003	0.12	0.013
*σ*_*l*_	0.04	0.004	0.22	0.02
*ρ*_12 _= 0.65, T_wald _= -7.3, LRT = 79, PM = -4.6 (p < 0.001 for all tests) The estimate of the common WSCV under the null is 0.034 (SE = 0.003) 95% CI for (θ_1_-θ_2_): (-0.12,-0.07)				

## Discussion

A comparison between the reproducibility of two measuring instruments using the same set of subjects leads naturally to a comparison of two dependent indices. In this paper, several procedures are developed for testing equality of two dependent within-subject coefficient of variations computed from the same sample of subjects. We proposed two approaches; one is likelihood based (LRT, Wald, and Score test), while the other is regression based approach (extension of Pitman-Morgan). We assessed the powers and the empirical levels of significance of these methods via extensive Monte Carlo simulations. It is shown that the relatively simple Wald's test (WT) is as powerful as the likelihood ratio test (LRT) and that both have consistently greater power than the score test. A simple procedure based on results due to Pitman [1] and Morgan [2] is also developed and shown to be as powerful as the likelihood based tests.

We illustrated the proposed methodologies with the analyses of data from two biomedical studies. The majority of microarray reproducibility and cross-platform agreement studies use Pearson's correlation, as an index of reproducibility and agreement, which would not be an appropriate measure of reproducibility. Because of the large heterogeneity among the genes in the data set, the intra-class correlation coefficient as an index of reproducibility of the platform would also not be an appropriate index of reliability as highly heterogeneous populations artificially produces high reliability index. Therefore, WSCV should be used as an index of reproducibility. In addition, the methodology presented in this paper overcomes the difficulty noted by Tan et al. [14] in which the authors state that "*Dependence between the datasets would confound any inferences we could make about the differences in correlations. ... determination whether differences in correlation were statistically significant could not be made*". In this paper, we have used the within- gene coefficient of variation as a measure of reproducibility of a specific platform. Therefore, a comparison across platforms leads naturally to a comparison of two dependent within-subject coefficients of variation.

Two issues need to be discussed in this section. The first is related to the nature of the data to be analyzed while the other is related to situations when the assumed underlying model generating the data deviates from the normal distribution.

First, a frequently occurring question in the planning of biomedical investigations is whether to measure the response or the trait of interest on a continuous scale (e.g. gene expressions; systolic blood pressures etc.) or dichotomous scale (e.g. highly expressed gene vs. low expressed genes; hypertensive vs. normtensive etc.). In the case of two measuring devices and two dichotomous responses, the most commonly used measure of test-retest reliability or agreement is the kappa coefficient introduced in [35]. Donner and Eliasziw [36] and more recently Shoukri and Donner [37] cautioned against dichotomizing traits measured on the continuous scales. They demonstrated that the loss in the efficiency in estimation of the reliability coefficient can be severe. The conclusion is that for naturally dichotomous traits (e.g. affected vs. not affected) one can use kappa to assess the test-re-test reliability, while for continuous traits the methods presented in this paper would be more appropriate.

Second, it should be noted that the inference procedures discussed in this paper (except the PM test) are likelihood based and their statistical properties may not be appropriate in small samples. The difficulty is that the sampling distribution of a test statistics is unknown. Alternatively, one may use the bootstrap technology to estimate the sampling distributions of the test statistics. When the data are hierarchical in nature with variance covariance matrix Σ as shown in (2), one may use model-based approach to generate bootstrap samples [38], which is achieved by sampling subjects with replacement and estimate the coefficients of variations and hence their empirical sampling distributions. There is already a rich class of bootstrap methods for clustered data in the literature but there is an absence of detailed theoretical results on the properties of these methods [39]. Gaining insight into bootstrapping clustered data for all these methods and draw comparison to our proposed likelihood based approach warrants serious investigation and is beyond the scope of this paper.

## Conclusion

Comparison of reproducibility or reliability of measurement devices or methods on the same set of subjects comes down to comparison of dependent reliability or reproducibility parameters. Testing the equality of two dependent WSCV has not been dealt with in the statistical literature. The presented methodology overcomes the difficulty noted by data analysts that the issue of dependence when ignored, would confound the inference on measures of reliability or reproducibility. It should also be emphasized that when comparison among platforms reliability indices the ICC is not an appropriate measure of reliability due to the large heterogeneity among the genes. Because the magnitude of the ICC depends on the degree of heterogeneity among the subjects it is not an appropriate index of reproducibility. We therefore recommend the WSCV in similar settings.

The LRT and WT procedures presented in Section 2 may also be extended in a straightforward manner to compare more than two platforms (methods, labs, or measurement devices). A further advantage of the LRT in this context is that it may easily be extended to deal with the case of an unequal number of replicates for each platform.

The codes developed (in MATLAB) can be used to do power calculations for planning a reproducibility study when comparing two methods (or devices), and can be obtained on request from the authors.

## APPENDIX

Elements of Fisher's information matrix (*m*_1 _= *m*_2 _= *m*)

$$\begin{array}{l} i_{33}=E\left(\frac{\partial^{2}l}{\partial \sigma_{1}^{2}\partial \sigma_{1}^{2}}\right)=\frac{n}{4\sigma_{1}^{4}}\left[2m+\frac{m^{2}}{w}\rho_{12}^{2}\right]\\ i_{34}=E\left(\frac{\partial^{2}l}{\partial \sigma_{1}^{2}\partial \sigma_{2}^{2}}\right)=-\frac{nm^{2}}{4\sigma_{1}^{2}\sigma_{2}^{2}w}\rho_{12}^{2}\\ i_{35}=E\left(\frac{\partial^{2}l}{\partial \sigma_{1}^{2}\partial \rho_{1}}\right)=-\frac{n\left(m-1\right)}{2\sigma_{1}^{2}}\left[\frac{1}{1-\rho_{1}}-\frac{1+\left(m-1\right)\rho_{2}}{w}\right]\\ i_{36}=i_{45}=0\\ i_{37}=E\left(\frac{\partial^{2}l}{\partial \sigma_{1}^{2}\partial \rho_{12}}\right)=-\frac{nm^{2}}{2w\sigma_{1}^{2}}\rho_{12}\\ i_{44}=E\left(\frac{\partial^{2}l}{\partial \sigma_{2}^{2}\partial \sigma_{2}^{2}}\right)=\frac{n}{4\sigma_{2}^{4}}\left[2m+\frac{m^{2}}{w}\rho_{12}^{2}\right]\\ i_{46}=E\left(\frac{\partial^{2}l}{\partial \sigma_{2}^{2}\partial \rho_{2}}\right)=-\frac{n\left(m-1\right)}{2\sigma_{2}^{2}}\left[\frac{1}{1-\rho_{2}}-\frac{1+\left(m-1\right)\rho_{1}}{w}\right]\\ i_{47}=E\left(\frac{\partial^{2}l}{\partial \sigma_{2}^{2}\partial \rho_{12}}\right)=-\frac{nm^{2}}{w}\frac{\rho_{12}}{2\sigma_{2}^{2}}\\ i_{55}=E\left(\frac{\partial^{2}l}{\partial \rho_{1}^{2}}\right)=\frac{n\left(m-1\right)}{2}\left[\frac{1}{{\left(1-\rho_{1}\right)}^{2}}+\left(m-1\right)\frac{{\left(1+\left(m-1\right)\rho_{2}\right)}^{2}}{w^{2}}\right]\\ i_{56}=E\left(\frac{\partial^{2}l}{\partial \rho_{1}\partial \rho_{2}}\right)=n{\left(m-1\right)}^{2}m^{2}\frac{\rho_{12}^{2}}{2w^{2}}\\ i_{57}=E\left(\frac{\partial^{2}l}{\partial \rho_{1}\partial \rho_{12}}\right)=-\frac{n\left(m-1\right)m^{2}\rho_{12}}{w}\left[1+\left(m-1\right)\rho_{2}\right]\\ i_{66}=E\left(\frac{\partial^{2}l}{\partial \rho_{2}^{2}}\right)=\frac{n\left(m-1\right)}{2}\left[\frac{1}{{\left(1-\rho_{2}\right)}^{2}}+\left(m-1\right)\frac{{\left(1+\left(m-1\right)\rho_{1}\right)}^{2}}{w^{2}}\right]\\ i_{67}=E\left(\frac{\partial^{2}l}{\partial \rho_{2}\partial \rho_{12}}\right)=-n\left(m-1\right)\frac{m^{2}\rho_{12}}{w}\left[1+\left(m-1\right)\rho_{1}\right]\\ i_{77}=E\left(\frac{\partial^{2}l}{\partial \rho_{12}^{2}}\right)=\frac{nm^{2}}{w}\left[1+2\rho_{12}^{2}\frac{m^{2}}{w^{2}}\right], \end{array}$$

The matrix *I*_22 _is therefore given by:

$$I_{22}=\left[\begin{array}{ccccc} i_{33} & i_{34} & i_{35} & 0 & i_{37}\\ & i_{44} & 0 & i_{46} & i_{47}\\ & & i_{55} & i_{56} & i_{57}\\ & & & i_{66} & i_{67}\\ & & & & i_{77} \end{array}\right]$$

## Competing interests

The authors declare that they have no competing interests.

## Authors' contributions

MMS conceived of the study problem and derived the analytical results. DC conducted the simulations and analyzed the data. All authors contributed to the writing of the manuscript, and approved its final format.

## Pre-publication history

The pre-publication history for this paper can be accessed here:


